# Complete genome sequence of *Bacillus velezensis* DDW3, an endophyte isolated from the leaves of *Matricaria chamomilla*

**DOI:** 10.1128/mra.00116-26

**Published:** 2026-05-18

**Authors:** Yusha Du, Jinbai Duan, Yiren Xu, Jiatong Zhang, Xiujuan Gan, Kangwei Xie, Xingyong Yang

**Affiliations:** 1 College of Pharmacy, Chengdu University 74707, Chengdu, China; University of Strathclyde, Glasgow, United Kingdom

**Keywords:** genome, endophyte, *Bacillus velezensis*, *Matricaria chamomilla*

## Abstract

The endophyte *Bacillus velezensis* strain DDW3 was isolated from the leaves of *Matricaria chamomilla*. Here, we report the complete genome sequence of strain DDW3, which consists of a single circular chromosome measuring 4,026,014 bp with a guanine-cytosine content of 46.5%.

## ANNOUNCEMENT

The genus *Bacillus* comprises over 228 species, including *B. subtilis*, *B. cabrialesii*, *B. tequilensis*, *B. altitudinis*, *B. mojavensis*, and *B. velezensis*. Many of these species exhibit significant potential in antagonizing plant pathogens and producing bioactive compounds (1). Notably, *B. velezensis* contributes to agriculture and medicine due to its anti-inflammatory, antibacterial, and antioxidant properties (2). These characteristics underscore the importance of exploring novel strains of *B. velezensis* to enhance their application potential.

In this study, we isolated the *Bacillus velezensis* strain DDW3 from surface-sterilized leaves of *Matricaria chamomilla* collected in Chengdu, China (30°39′39″N, 104°11′15″E). Sterilization involved 75% ethanol (1 min) and 2% sodium hypochlorite (3 min), followed by three sterile water rinses (3). After aseptic drying, the leaves were cut into small segments (approximately 5 × 5 mm) and placed on Luria-Bertani (LB) agar plates. Following incubation at 37°C for 24–48 h, colonies emerging from the leaf margins were streaked onto fresh LB agar plates and incubated at 37°C for 24 h to ensure purity. One isolate, designated DDW3, was selected based on its morphology and stored in 25% glycerol at −80°C (4, 5).

Genomic DNA was extracted from strain DDW3 using the Wizard Genomic DNA Purification Kit (Promega, USA) following the manufacturer’s instructions. Briefly, the strain was revived from glycerol stock (−80°C) by streaking onto LB agar and incubating at 37°C under aerobic conditions for 24  h. A single colony was then inoculated into LB broth and cultured under identical conditions until reaching the late-exponential growth phase, approximately 12 h. Cells were then harvested from the culture for genomic DNA extraction (6). Sequencing combined PacBio Sequel IIe SMRT and Illumina NovaSeq 6000 approach (7). Libraries were prepared with the SMRTbell Express Template Prep Kit 2.0 (PacBio, USA) and the Nextera DNA Flex Library Preparation Kit (Illumina, USA) according to the manufacturers’ protocols. Sequencing produced 57,242 PacBio HiFi reads (N50: 11,887 bp; average length: 11,758.15 bp) and 6,862,210 Illumina paired-end reads (2 × 150 bp). Illumina reads were trimmed with fastp v0.20.0 (8), and PacBio HiFi reads were processed with SMRT Link v11.0.

*De novo* assembly was conducted using PacBio HiFi reads with Canu v2.2 (9). Canu automatically detected and trimmed the overlapping ends by aligning the contig termini to circularize the sequence, and then rotated the genome to start at the *dnaA* gene, yielding a complete circular chromosome of 4,026,014 bp. The assembly was then polished with Illumina reads using Pilon v1.24 (10, 11), resulting in 46.5% guanine-cytosine (GC) content. The taxonomic identity of strain DDW3 was verified through whole-genome average nucleotide identity (ANI) analysis utilizing the EzBioCloud (database version 2025.04.21) online calculator with the OrthoANIu algorithm (12). The analysis revealed an average nucleotide identity of 97.86% between strain DDW3 and the type strain *Bacillus velezensis* (GCA_001267695.1), significantly exceeding the 95% species delineation threshold. The assembly was annotated using the National Center for Biotechnology Information (NCBI) Prokaryotic Genome Annotation Pipeline (PGAP) v6.10 (13), which identified 3,904 coding sequences, 86 tRNAs, and 27 rRNAs. Default parameters were used unless otherwise specified.

## Data Availability

The complete genome sequence of *Bacillus velezensis* DDW3, along with the raw Illumina reads (SRA: SRR35671782) and PacBio SMRT reads (SRA: SRR37048054), has been deposited in National Center for Biotechnology Information (NCBI) under BioProject PRJNA1335904 and BioSample SAMN52029460. The assembled genome is available under GenBank accession GCF_054165805.1. Supplementary annotation files from AntiSMASH are available at Zenodo: https://doi.org/10.5281/zenodo.18975213.
